# Correlation of high-resolution computed tomography and immunological bronchoalveolar lavage in interstitial lung disease at the onset of inflammatory rheumatic diseases: implications for diagnosis and therapeutic strategies

**DOI:** 10.1186/s13075-024-03371-0

**Published:** 2024-07-29

**Authors:** Tobias Hoffmann, Ulf Teichgräber, Martin Förster, Peter Oelzner, Claus Kroegel, Diane Renz, Tobias Weise, Joachim Böttcher, P. Christian Schulze, Gunter Wolf, Marcus Franz, Alexander Pfeil

**Affiliations:** 1 Department of Internal Medicine III, Jena University Hospital – Friedrich Schiller University Jena, Am Klinikum 1, 07747 Jena, Germany; 2 Institute of Diagnostic and Interventional Radiology, Jena University Hospital – Friedrich Schiller University Jena, Am Klinikum 1, 07747 Jena, Germany; 3 Department of Internal Medicine I, Jena University Hospital – Friedrich Schiller University Jena, Am Klinikum 1, 07747 Jena, Germany; 4 Institute of Diagnostic and Interventional Radiology, Department of Pediatric Radiology, Hannover Medical School, Carl-Neuberg-Str. 1, 30625 Hannover, Germany; 5 BioControl Jena GmbH, Jena, Germany

## Abstract

**Objectives:**

Inflammatory rheumatic diseases (IRD) are often associated with interstitial lung disease (ILD). The aim of the present study was to establish a correlation between the findings on HRCT and the immunological bronchoalveolar lavage (BAL).

**Methods:**

The study included 74 patients with newly diagnosed IRD and evidence of ILD on HRCT with the following pattern: ground-glass opacities (GGO), non-specific interstitial pneumonia (NSIP) and usual interstitial pneumonia (UIP). Patients with other HRCT pattern were excluded. No patient received any immunosuppressive therapy. In addition to HRCT, immunological BAL was performed and the American Thoracic Society clinical practice guideline were used to define BAL patterns (lymphocytic cellular pattern, neutrophilic cellular pattern, eosinophilic cellular pattern and unspecified pattern).

**Results:**

The main HRCT patterns were NSIP (47.3%), GGO (33.8%), and UIP (18.9%). BAL patterns showed the following distribution: 41.9% lymphocytic cellular pattern, 23.0% neutrophilic cellular pattern, 18.9% eosinophilic cellular pattern, and 16.2% unspecific cellular pattern. Placing these data in the context of the HRCT findings, the lymphocytic cellular BAL pattern (48%) was most commonly BAL pattern associated with GGO pattern in HRCT, whereas neutrophilic and lymphocytic cellular BAL patterns were the dominant feature in NSIP and UIP.

**Conclusion:**

In patients with new-onset IRD and ILD, inflammatory pulmonary changes are predominate, reflected by GGO on HRCT and a mainly lymphocytic cell profile in the immunological BAL. In NSIP or UIP on HRCT, the percentages of lymphocytes and neutrophils were higher in BAL fluid, representing a fibrotic component in addition to the inflammation. Consequently, patients with evidence of GGO on HRCT should primarily be treated with anti-inflammatory/immunosuppressive therapy, whereas in patients with NSIP and UIP a combination of anti-inflammatory and anti-fibrotic agents would be the appropriate treatment.

## Introduction

Inflammatory rheumatic disease (IRD) represent a group of immune-mediated inflammatory diseases, including inflammatory joint disease, connective tissue diseases (CTD), myositis, and vasculitis [1]. Interstitial lung disease (ILD) is one of the main organ manifestation ranging between 12 and 80% in IRD-patients [2]. In addition, IRD-ILD is associated with an increased mortality depending on the underlining IRD [3].

International guidelines currently recommend high-resolution computed tomography (HRCT) as the gold standard imaging modality for the evaluation of IRD-ILD [4–6]. In established IRD-ILD, HRCT often reveals a specific pattern like usual interstitial pneumonia (UIP), non-specific interstitial pneumonia (NSIP) or ground-glass opacities (GGO) [7]. Unfortunately, HRCT does not allow the differentiation between inflammatory and fibrotic changes of the lung. In addition, patients with new-onset IRD and ILD may not have the characteristic patterns that allow a diagnosis to be made with a high level of confidence using HRCT imaging alone.

Bronchoalveolar lavage (BAL) is a minimally invasive technique for obtaining fluid from the pulmonary bronchoalveolar lining for diagnostic purposes. In patients with suspected ILD, the recommended diagnostic examinations performed on BAL fluid include differential cell count, microbiological studies, and malignant cell cytology laboratory testing [1]. Based on these cell patterns, some ILDs (e.g. sarcoidosis, pulmonary alveolar proteinosis) can be diagnosed by BAL [8, 9]. However, the role of BAL for the differentiation between inflammation and fibrosis in IRD-ILD is less clear.

So far, there are no data on a possible association between HRCT and BAL patterns in patients with IRD and ILD. Our study addressed this question.

## Patients and methods

The study included 74 newly diagnosed IRD-patients (61 women and 13 men) with CTD (*N* = 46), myositis (*N* = 12), vasculitis (*N* = 10), and rheumatoid arthritis (RA; *N* = 6). Details regarding baseline characteristics see Table 1.

**Table 1:** Baseline characteristics of ILD-group

	CTD	Myositis	Vasculitis	RA
**Number of patients**	*N* = 46	*N* = 12	*N* = 10	*N* = 6
**Age** mean ± standard deviation	62.1 ± 14.5 years	60.1 ± 9.7 years	61.7 ± 10.7 years	63.0 ± 10.9 years
**Gender**	Women *N* = 42 (91.3%) Men *N* = 4 (8.7%)	Women *N* = 8 (66.7%) Men *N* = 4 (33.3%)	Women *N* = 6 (60%) Men *N* = 4 (40%)	Women *N* = 5 (83.3%) Men *N* = 1 (16.7%)
**IRD**	SLE: *N* = 9 Sjogren´s disease: *N* = 7 SSc: *N* = 25 MCTD: *N* = 5	Dermatomyositis: *N* = 5 Polymyositis: *N* = 1 Anti Jo1-syndrome: *N* = 6	EGPA: *N* = 5 GPA: *N* = 2 MPA: *N* = 3	*N* = 6
**Immunological laboratory findings**	ANA positive: *N* = 46 Scl 70-antibody positive: *N* = 13 Ro-antibody positive: *N* = 8 dsDNS-antibody positive: *N* = 7 Centromer-antibody positive: *N* = 5 U1RNP-antibody positive: *N* = 4 PM-Scl-antibody positive: *N* = 2 Tif1g-antibody positive: *N* = 1	ANA positive: *N* = 7 Anti Jo1-antibody positive: *N* = 6 MDA5-antibody positive: *N* = 2 PL-12-antibody positive: *N* = 1 PL-7-antibody positive: *N* = 1	MPO-antibody positive: *N* = 3 PR3-antibody positive: *N* = 2	Anti-CCP-antibody positive: *N* = 5 RF-positive: *N* = 4

ANA = Antinuclear antibody; CTD = Connective tissue disease; EGPA: Eosinophilic granulomatosis with polyangiitis; GPA = *Granulomatosis* with polyangiitis; IRD = Inflammatory rheumatic disease; MPA = Microscopic polyangiitis; RA = Rheumatoid arthritis; SLE = *Systemic lupus* erythematosus; SSc = Systemic sclerosis

If the pulmonary function test (PFT) showed a low carbon monoxide diffusing capacity (DLCO) < 80% which is considered to be the earliest abnormality on PFT in patients with ILD, a HCRT was performed. Patients were included in our study if the following HCRT (using the standard protocol of the manufacturers) showed ground-glass opacities (GGO), non-specific interstitial pneumonia (NSIP), and/or usual interstitial pneumonia (UIP) [7]. There was no minimum amount of fibrosis required for a patient to be classed as having ILD. Only the presence of HRCT patterns was evaluated. All HRCT scans were read by a radiologist and a rheumatologist. In the case of ambiguous findings, the final decision was made by a second radiologist. Immunosuppressive or immunomodulatory treatment (including glucocorticoids), pre-existing pulmonary diseases, current or former smoking as well as infection(s) at the time point of immunological BAL were exclusion criteria.

### Immunological bronchoalveolar lavage

All patients underwent an immunological BAL. The procedure itself as well as the handling and processing of the BAL fluid were performed according to the recommendations of the American Thoracic Society (ATS) clinical practice guideline [10]. Besides microbiological studies and cytopathology, a differential cell count analysis was done. The interpretation of BAL nucleated immune cell patterns was also based on the ATS clinical practice guideline using the following definitions [10]:

- Lymphocytic cellular pattern > 15% lymphocytes.
- Neutrophilic cellular pattern > 3% neutrophils.
- Eosinophilic cellular pattern > 1% eosinophils.

The above mentioned pattern were complemented by unspecific cellular pattern (< 15% lymphocytes, < 3% neutrophils, < 1% eosinophils) recommended as modified ATS.

In detail, the ATS classification was simplified into just four categories: Lymphocytic cellular pattern, neutrophilic cellular pattern, eosinophilic cellular pattern, and unspecified pattern, called modified ATS. For each patient, the percentages of lymphocytes, neutrophils, and eosinophils are compared to set thresholds (15% for lymphocytes, 3% for neutrophils, and 1% for eosinophils). The category with the highest positive difference is chosen. If there is no positive difference, the patient is labeled as “Unspecified cellular pattern”.

### Statistical analysis

Data collection and documentation was carried out using Microsoft Excel^®^ (Microsoft Windows, Redmond Washington, USA). Descriptive data analysis and data processing were performed with the Python programming language (version 3.10.0) as well as the additional packages Numpy (version 1.22.3), Pandas (version 1.4.1), and Scipy (version 1.8.0).

During data processing, patients were classified into BAL patterns according to the ATS guideline [10]. However, the ATS classification was restricted to just three groups: Lymphocytic cellular pattern (‘Lym’), neutrophilic cellular pattern (‘Neu’), and eosinophilic cellular pattern (‘Eos’).

Therefore, the classification was referred to as ‘modified ATS’ (‘modATS’) in the following. The classification procedure was as followed:

1. Respective thresholds as obtained from ATS are subtracted from the fractions of lymphocytes (Lym: 0.15), neutrophils (Neu: 0.03), and eosinophils (Eos: 0.01).
2. Patients are assigned to the respective BAL pattern (‘Lym’, ‘Neu’, ‘Eos’) with the greatest positive difference value.
3. If no difference greater than 0 is observed, allocation to the category ‘Unspecified’ was made.

The respective statistical significance level of group differences between BAL pattern (modified ATS pattern) and HRCT pattern were calculated using Fisher’s exact test (scipy.stats.fisher_exact). *P*-values < 0.05 were considered as significant.

## Results

### HRCT

For the total study cohort, the main HRCT patterns were NSIP (47.3%) and GGO (33.8%), followed by UIP (18.9%) (Table 2). NISP was also the leading finding in patients with CTD (45.7%) and myositis (75.0%), whereas GGO was most frequently seen in vasculitis (60.0%) and RA (50.0%). Further, UIP was the least frequently detected pattern (CTD 19.5%, myositis 25.0%, vasculitis 20.0% and RA 0%) in the initial diagnosis of IRD with ILD.

**Table 2:** HCRT patterns in different IRD

	CTD *N* = 46	Myositis *N* = 12	Vasculitis *N* = 10	RA *N* = 6	Total *N* = 74
**GGO**	34.8% (*N* = 16)	0% (*N* = 0)	60.0% (*N* = 6)	50.0% (*N* = 3)	33.8% (*N* = 25)
**NSIP**	45.7% (*N* = 21)	75.0% (*N* = 9)	20.0% (*N* = 2)	50.0% (*N* = 3)	47.3% (*N* = 35)
**UIP**	19.5% (*N* = 9)	25.0% (*N* = 3)	20.0% (*N* = 2)	0% (*N* = 0)	18.9% (*N* = 14)

CTD = Connective tissue disease; GGO = Ground-glass opacity; IRD = Inflammatory rheumatic disease; NISP = Non-specific interstitial pneumonia; RA = Rheumatoid arthritis; UIP = Usual interstitial pneumonia

### Immunological BAL pattern

The total cell count was similar between all IRD groups (CTD 2.4 × 10^5^, myositis 3.1 × 10^5^, and vasculitis 3.4 × 10^5^) (Table 3). Compared to ATS reference values, lymphocytes (*ATS reference value 10–15%*) were increased in RA (27.0%), myositis (26.8%), CTD (20.8%), and vasculitis (17.2%). Neutrophils (*ATS reference value < 3%*) were elevated in CTD (6.6%) and myositis (8.5%) and within the normal range in vasculitis (2.7%). Regarding eosinophils (*ATS reference value < 5%*) no increased mean values were observed for CTD (3.2%), myositis (3.5%) and RA (0.7%) with exception of vasculitis (7.1%).

**Table 3:** Immunological BAL cell patterns in different IRD

	CTD *n* = 46	Myositis *n* = 12	Vasculitis *n* = 10	RA *n* = 6	Total *n* = 74	Reference values according to ATS clinical practice guideline [10]
Cell count (total) mean ± SD	2.4 × 10^5^ ± 1.7 × 10^5^	3.1 × 10^5^ ± 1.0 × 10^5^	3.4 × 10^5^ ± 2.2 × 10^5^	1.6 × 10^5^ ± 1.7 × 10^5^	2.6 × 10^5^ ± 1.0 × 10^5^	-
Alveolar macrophages mean ± SD	67.9 ± 20.5%	58.0 ± 17.0%	71.8 ± 20.9%	67.8 ± 19.0%	66.8 ± 20.0%	> 85%
Lymphocytes (CD4+/CD8+) mean ± SD	20.8 ± 15.9%	26.8 ± 16.1%	17.2 ± 16.2%	27.0 ± 20.5%	21.8 ± 16.3%	10–15%
Neutrophils mean ± SD	6.6 ± 8.1%	8.5 ± 6.9%	2.7 ± 2.4%	3.3 ± 2.7%	6.1 ± 7.2%	< 3%
Eosinophils mean ± SD	3.2 ± 4.1%	3.5 ± 5.8%	7.1 ± 14.6%	0.7 ± 0.5%	3.6 ± 6.6%	< 5%

ATS = American Thoracic Society; CTD: Connective tissue disease; RA = Rheumatoid arthritis; SD = Standard deviation

The main modified ATS BAL pattern was a lymphocytic cellular pattern (41.9%), followed by neutrophilic cellular pattern (23.0%), eosinophilic cellular pattern (18.9%), and unspecified cellular pattern (16.2%) (Table 4). The same results were observed for CTD, myositis, and RA with an increased presence of lymphocytes. In patients with vasculitis, the eosinophilic cellular pattern was predominant.

**Table 4:** BAL cell pattern in different IRD according to the ATS clinical practice guideline [10]

Modified ATS guidelines	CTD *N* = 46	Myositis *N* = 12	Vasculitis *N* = 10	RA *N* = 6	Total *N* = 74
Lymphocytic cellular pattern	39.1% (*N* = 18)	58.4% (*N* = 7)	30.0% (*N* = 3)	50.0% (*N* = 3)	41.9% (*N* = 31)
Neutrophilic cellular pattern	26.1% (*N* = 12)	33.3% (*N* = 4)	0% (*N* = 0)	16.7% (*N* = 1)	23.0% (*N* = 17)
Eosinophilic cellular pattern	19.6% (*N* = 9)	8.3% (*N* = 1)	40.0% (*N* = 4)	0% (*N* = 0)	18.9% (*N* = 14)
Unspecific cellular pattern	15.2% (*N* = 7)	0% (*N* = 0)	30.0% (*N* = 3)	33.3% (*N* = 2)	16.2% (*N* = 12)

ATS = American Thoracic Society; CTD: Connective tissue disease; RA = Rheumatoid arthritis

### Immunological BAL patterns in correlation with HRCT pattern

The highest cell count (2.7 × 10^5^) was found for GGO compared with NSIP (2.6 × 10^5^) and UIP (2.4 × 10^5^) and the highest neutrophil count was observed for NSIP (7.8%). Compared to the reference values according to the ATS clinical practice guideline, in GGO lymphocytes (19.7%, *ATS reference value: 10–15%*) were increased by normal neutrophils and eosinophils. For NSIP and UIP the lymphocytes (NSIP 24.0% versus UIP 20.0%, *ATS reference value: 10–15%*) and neutrophils (NSIP 7.8% versus UIP 6.9%, *ATS reference value: <3%*) were increases by a normal range of eosinophiles (Table 5).

**Table 5:** BAL cell pattern depending on HRCT pattern

	GGO *N* = 25	NSIP *N* = 35	UIP *N* = 14	Reference values according to ATS clinical practice guideline [10]
Cell count (total) mean ± SD	2.7 × 10^5^ ± 2.2 × 10^5^	2.6 × 10^5^ ± 1.5 × 10^5^	2.4 × 10^5^ ± 1.3 × 10^5^	-
Alveolar macrophages mean ± SD	73.2 ± 20.4%	61.6 ± 20.9%	68.4 ± 13.1%	> 85%
Lymphocytes (CD4+/CD8+) mean ± SD	19.7 ± 17.3%	24.0 ± 17.7%	20.0 ± 9.6%	10–15%
Neutrophils mean ± SD	3.3 ± 3.0%	7.8 ± 9.1%	6.9 ± 6.3%	< 3%
Eosinophils mean ± SD	3.0 ± 9.5%	4.1 ± 4.5%	3.5 ± 5.2%	< 5%

ATS = American Thoracic Society; BAL = Bronchoalveolar lavage; GGO = Ground-glass opacity; HRCT = High-resolution computed tomography; NISP = Non-specific interstitial pneumonia; UIP = Usual interstitial pneumonia

**Fig. 1:**
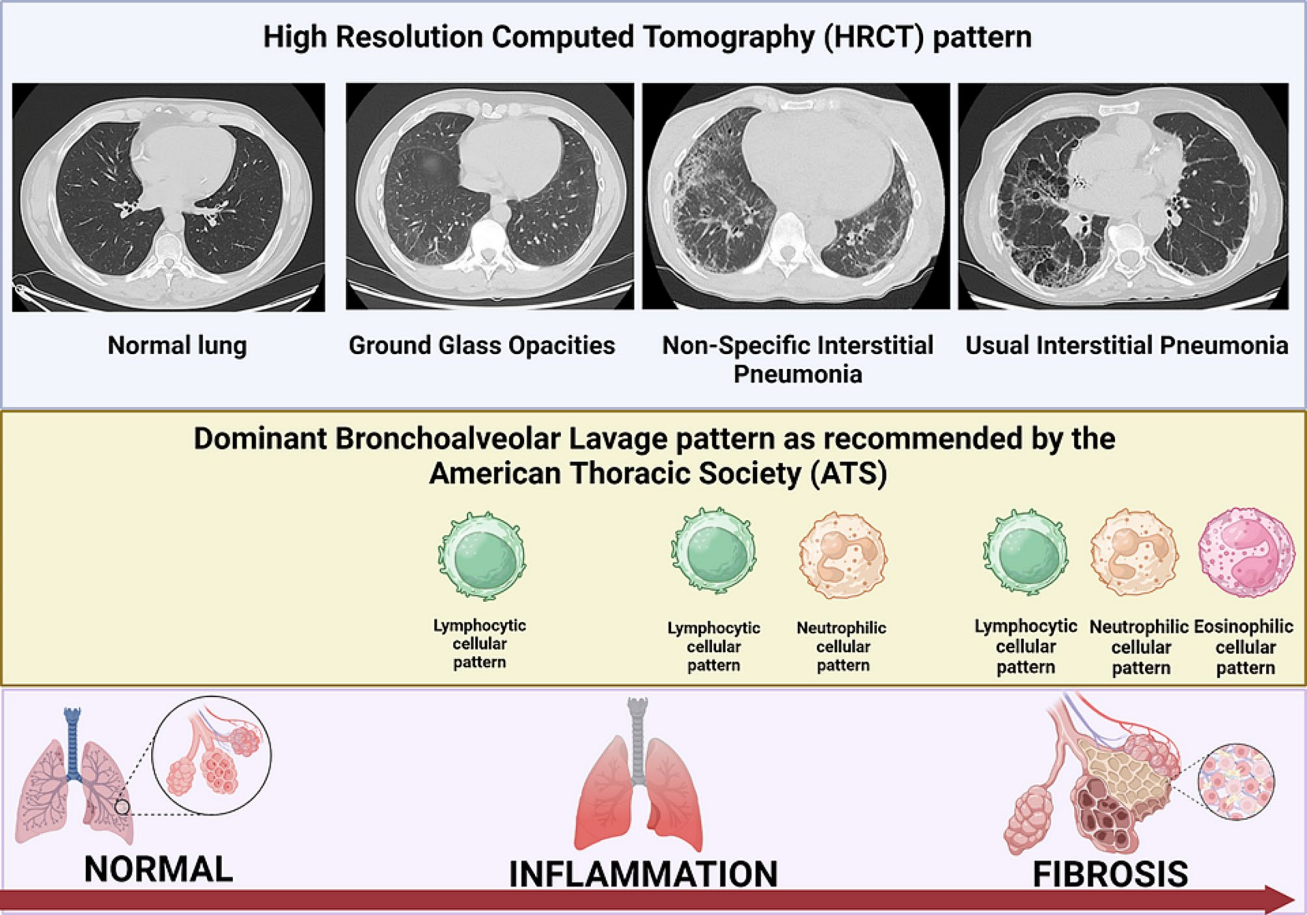
Dominant BAL patterns as recommended by the ATS in correlation to HRCT patterns “Created with BioRender.com” (ATS = American Thoracic Society; BAL = Bronchoalveolar lavage; HRCT = High-resolution computed tomography)

Further, the lymphocytic pattern (48.0%) was the most common BAL pattern seen in the patients with GGO. Neutrophilic and lymphocytic cellular patterns (34.3%) were the frequent BAL pattern in NSIP (Table 6). The ratio of HRCT pattern ‘NSIP/UIP’ to ‘GGO’ was significantly higher (p = 0.040) in the BAL pattern group neutrophiles than in the BAL pattern group lymphocytes/eosinophils/unspecific (see Fig. 1). The ratio of HRCT pattern ‘NSIP/UIP’ to ‘GGO’ showed no significant (*p* = 0.467) differences between BAL pattern group lymphocytes versus BAL pattern group neutrophiles /eosinophils/unspecific.

**Table 6:** BAL cell patterns according to the American thoracic society (ATS) guideline [10] versus HRCT patterns

Modified ATS guidelines	HRCT pattern			
	GGO *N* = 25	NSIP *N* = 35	UIP *N* = 14	Total *N* = 74
Lymphocytic cellular pattern	48.0% (*N* = 12)	34.3% (*N* = 12)	50.0% (*N* = 7)	41.9% (*N* = 31)
Neutrophilic cellular pattern	8.0% (*N* = 2)	34.3% (*N* = 12)	21.4% (*N* = 3)	23.0% (*N* = 17)
Eosinophilic cellular pattern	16.0% (*N* = 4)	20.0% (*N* = 7)	21.4% (*N* = 3)	18.9% (*N* = 14)
Unspecific cellular pattern	28.0% (*N* = 7)	11.4% (*N* = 4)	7.2% (*N* = 1)	16.2% (*N* = 12)

ATS = American Thoracic Society; BAL = Bronchoalveolar lavage; HRCT = High-resolution computed tomography

## Discussion

IRD-ILD has increasingly become the focus of clinical and scientific considerations in recent years, also in the light of new available therapeutic options.

To the best of our knowledge, the present study is the first to evaluate HRCT and immunological BAL patterns in a population of newly diagnosed, immunosuppressive-naïve patients with IRD- ILD.

HRCT is currently the gold standard for the detection of IRD-ILD [4–6]. Typical HRCT patterns in IRD-ILD include GGO, NSIP, and UIP. In the literature, GGO has been described as being associated with alveolitis [11], whereas NSIP and UIP patterns are the main HRCT features in pulmonary fibrosis [12, 13].

In the first step of our investigation, the HRCT patterns were assigned to the different IRDs. The data showed that the HRCT patterns of GGO, NSIP, and UIP are not specific for a disease entity of IRD-ILD.

In a second step, we evaluated the IRD-ILD and HRCT patterns taking into account the results of the immunological BAL classified according to the ATS criteria. The lymphocytic cellular BAL pattern was most commonly associated with GGO, and the neutrophilic and lymphocytic cellular BAL findings with NSIP and UIP patterns, respectively.

For the total study cohort, the main HRCT patterns were NSIP (47.3%), GGO (33.8%), and UIP (18.9%), whereas no HRCT pattern was specific for any IRD subtype at the time of the diagnosis. Oliveira et al. reported 60% NSIP and 36% UIP as dominant HRCT pattern [14] with a mean duration of IRD of nine years reflecting a prolonged course of ILD [15]. In our study, only patients with the initial diagnosis of IRD were included, who were diagnosed with lung involvement by comprehensive screening according to the algorithm developed by Hoffmann et al. [2]. Therefore, at the time of the initial diagnosis of IRD with ILD, a different distribution of HRCT patterns with GGO predominating was found. This observation is supported by data from Shah et al., who also demonstrated GGO as the most common HRCT pattern (66%) at initial diagnosis of ILD in patients with SSc [16]. When HRCT is performed at a very early stage or at the initial diagnosis of IRD, GGO is the predominant primary HRCT pattern. As ILD progresses, fibrotic HRCT changes with the NSIP and UIP patterns come to the fore [17].

Given the described changes in HRCT patterns over time from GGO to fibrotic patterns such as NSIP and UIP, it is of great interest how the HRCT patterns can be classified in relation to immunological BAL. It is generally accepted, that this technique can be used for diagnosing lung diseases and various ILD such as sarcoidosis [5, 18–20]. Our study results demonstrated that this cannot be transferred to IRD, as no BAL pattern was specific for IRD. Therefore, the immunological BAL is not an appropriate technique for diagnosing IRD.

However, we revealed in our study in patients at the onset of IRD and signs of ILD other clinically relevant findings such as a predominantly inflammatory cellular pattern with increased lymphocytes and neutrophils in the BAL differential cell profile in patients with GGO on HRCT. Orlandi et al. described the association of a lymphocytic cellular pattern with inflammatory alveolitis [20]. It is possible that this disease represents the starting point of IRD-ILD which, if left untreated, progresses longitudinally to fibrosis [21] and can subsequently be classified as NSIP and UIP patterns on HRCT.

Furthermore, in the NSIP pattern, interstitial inflammation can be detected histologically in addition to fibrosis [22]. This explains our finding of a combined neutrophilic and lymphocytic cellular pattern (both 34.3%) in the immunological BAL. This is corroborated by the data of an older study by Silver et al. in patients with SSc showing that the presence of neutrophils was associated with more advanced radiographic features of interstitial fibrosis in patients with disease of more than one year’s duration [21].

The results of our study have relevant therapeutic implications. In patients with initially diagnosed IRD and ILD, inflammatory changes in the lung dominate, represented by GGO on HRCT and the lymphocytic cellular pattern on immunological BAL. The treatment of choice should therefore be anti-inflammatory drugs, as ILD-patients with a BAL lymphocytosis showed a good response to anti-inflammatory treatment with glucocorticoids or in combination with immunosuppressive drugs (e.g., azathioprine, cyclosporine A, tacrolimus or cyclophosphamide) [23]. In the case of NSIP or UIP on HRCT, a lymphocytic and neutrophilic cell pattern can be detected in the immunological BAL. This suggests a fibrotic component in addition to the inflammation. As a result, a combined anti-inflammatory and anti-fibrotic therapy should be initiated in these patients [24–27].

A limitation of our investigation is the descriptive nature of the data. However, our study includes one of the largest cohorts of patients with ILD at the time of initial diagnosis of IRD who were not receiving immunosuppressive therapy. Therefore, our findings provide insights into the cellular immunological processes in IRD-ILD, which may have direct implications for the treatment of pulmonary involvement in patients with IRD. In this context, smokers and patients with an infection were also excluded, so that there is no effect of these two potential confounders on the data. Consequently, a large proportion of patients was excluded. Further, the descriptive study design can potentially influence the selection of the patients.

## Conclusion

In summary, our study revealed that there is no correlation between HRCT or cellular patterns in the immunological BAL to IRD-ILD. Therefore, an IRD with pulmonary involvement cannot be diagnosed solely on the basis of HRCT or the cellular pattern in the immunological BAL. Rather, we were able to demonstrate an association between GGO on HRCT with a lymphocytic cell pattern in the immunological BAL as a sign of an immunological inflammation of the alveoli, whereas NSIP and UIP were associated with a neutrophilic and lymphocytic cell pattern indicating an inflammation or fibrosis of the lung tissue.

## Data Availability

No datasets were generated or analysed during the current study.

## Declarations

### Patient and public involvement

Patients and/or the public were not involved in the design, or conduct, or reporting, or dissemination plans of this research.

## Abbreviations

ATS: American Thoracic Society
BAL: Bronchoalveolar lavage
GGO: Ground-glass opacities
HRCT: High-resolution computed tomography
ILD: Interstitial lung disease
IRD: Inflammatory rheumatic diseases
NSIP: Non-specific interstitial pneumonia
RA: Rheumatoid arthritis
SLE: Systemic lupus erythematosus
SSc: Systemic sclerosis
UIP: Usual interstitial pneumonia
